# The role of diagnostic laparoscopy in gynecology: O papel da laparoscopia diagnóstica em ginecologia

**DOI:** 10.1590/1516-3180.2014.00241501

**Published:** 2015-10-09

**Authors:** Raquel Togni, Cristina Laguna Benetti-Pinto, Daniela Angerame Yela

**Affiliations:** I Medical Student, School of Medical Sciences, Universidade Estadual de Campinas (Unicamp), Campinas, São Paulo, Brazil.; II MD, PhD. Professor, Department of Gynecology and Obstetrics, School of Medical Sciences, Universidade Estadual de Campinas (Unicamp), Campinas, São Paulo, Brazil.

**Keywords:** Laparoscopy, Infertility, Pelvic pain, Endometriosis, Endoscopy

## Abstract

**CONTEXT AND OBJECTIVES:**

Laparoscopy is a diagnostic method that is currently becoming consolidated for therapeutic use. It consists of endoscopically viewing the abdominal cavity. The aim here was to evaluate the indications for diagnostic videolaparoscopy and the intraoperative findings in an endoscopic gynecology clinic at a tertiary-level hospital over the last five years.

**DESIGN AND SETTING:**

Retrospective descriptive study on all diagnostic videolaparoscopy procedures of the last five years carried out in the endoscopic gynecology clinic of a tertiary-level hospital.

**METHODS:**

The medical records of 618 women who underwent diagnostic laparoscopy between 2008 and 2012 were analyzed. The clinical characteristics of these women, the indications for videolaparoscopy and the intraoperative findings were evaluated.

**RESULTS:**

The women’s mean age was 32 ± 6.4 years. Most of the women had already undergone at least one previous operation (60%), which was most frequently a cesarean. The indications for performing videolaparoscopy were infertility in 57%, chronic pelvic pain in 27% and others (intrauterine device, adnexal tumor, ectopic pregnancy or pelvic inflammatory disease) in 16%. The main laparoscopic findings were tubal alterations in the group with infertility (59.78%) and peritoneal alterations in the group with chronic pelvic pain (43.54%).

**CONCLUSION:**

The main indications for videolaparoscopy in gynecology were infertility and chronic pelvic pain. However, in most procedures, no abnormalities justifying these complaints were found.

## RESUMO

**CONTEXTO E OBJETIVOS:**

A laparoscopia é um método diagnóstico que atualmente se consolida como terapêutico e se caracteriza pela visão endoscópica da cavidade abdominal. O objetivo foi avaliar as indicações de videolaparoscopia diagnóstica e os achados intraoperatórios no serviço de videolaparoscopia ginecológica de um hospital terciário nos últimos cinco anos.

**DESENHO E LOCAL:**

Estudo retrospectivo descritivo com todos os procedimentos videolaparoscópicos diagnósticos dos últimos cinco anos realizados no serviço de ginecologia endoscópica de um hospital terciário.

**MÉTODOS:**

Foram analisados 618 prontuários de mulheres submetidas à laparoscopia diagnóstica entre os anos de 2008 a 2012. Foram avaliadas as características clínicas dessas mulheres, bem como as indicações da videolaparoscopia e os achados intraoperatórios.

**RESULTADOS:**

As mulheres tinham em média 32 ± 6.4 anos. A maioria das mulheres já tinha feito pelo menos uma cirurgia anterior (60%), sendo que a mais frequente foi a cesárea. As indicações para a realização da videolaparoscopia foram 57% por infertilidade, 27% por dor pélvica crônica e 16% outras (dispositivo intrauterino, tumor anexial, gravidez ectópica e doença inflamatória pélvica). Os principais achados laparoscópicos foram alterações tubárias no grupo com infertilidade (59.78%) e alterações peritoneais no grupo com dor pélvica (43.54%).

**CONCLUSÃO:**

As principais indicações da videolaparoscopia em ginecologia são infertilidade e dor pélvica crônica; mas na maior parte dos procedimentos não foram encontradas alterações que justifiquem essas queixas.

## INTRODUCTION

Laparoscopy is a diagnostic method that has become consolidated for therapeutic use. It consists of endoscopic viewing of the abdominal cavity by means of distention provided by artificial pneumoperitoneum. The first description of laparoscopy was by Ott and Kelling in 1901.^1^

Diagnostic laparoscopy is traditionally carried out in an operating theatre under general anesthesia. The procedure takes between 20 and 30 minutes and the patients are usually discharged from the hospital on the same day. Although laparoscopy is a simple technique, it is not free from complications, such as infections, hemorrhage and injuries of other abdominal-pelvic organs (bowel or bladder, for example). It has been shown that laparoscopy can diagnose pelvic pathological conditions in approximately 50% of the cases.^2^

The indications for diagnostic laparoscopy are infertility, chronic pelvic pain, pelvic tumors, pelvic inflammatory disease, genital tuberculosis and ectopic pregnancy.^1^ The present study evaluated the indications for diagnostic laparoscopy in a university hospital over the last five years.

## OBJECTIVE

To evaluate the indications for diagnostic laparoscopy and the intraoperative findings in the endoscopic gynecology clinic of a tertiary-level hospital in Campinas over the last five years.

## METHODS

A retrospective descriptive study was conducted in the Department of Gynecology and Obstetrics of a tertiary-level hospital in Campinas. A total of 618 medical records, from all diagnostic laparoscopy procedures performed in the endoscopic gynecology clinic between 2008 and 2012, were analyzed. The clinical characteristics of these women, the indications for laparoscopy and the intraoperative findings were evaluated. The protocol for this study was approved by our institution’s Review Board, under number 342.431/2013.

Descriptive analysis (frequencies, means and standard deviations) was performed on the categorical variables. To evaluate associations between the variables, the Kruskal-Wallis test was used. The significance level for statistical tests was 5%. SAS version 9.2 was used.

## RESULTS

The women’s mean age was 32 ± 6.4 years and their mean body mass index (BMI) was 25.6 ± 4.8 kg/m^2^. Among these women, 3% were menopausal and 39% were nulligravid. Diabetes was presented by 1.4%, hypertension by 3.8% and hypothyroidism by 3.4%, and 6.6% were smokers. Most of the women had already undergone at least one operation previously (60%), which was most frequently a cesarean.

The indications for laparoscopy were infertility in 57%, chronic pelvic pain in 27% and others (intrauterine device, adnexal tumor, ectopic pregnancy or pelvic inflammatory disease) in 16%.

The mean age of the women presenting infertility was 32 ± 4.4 years and, among the women with chronic pelvic pain, it was 34 ± 6.9 years (P = 0.04). There was no significant difference in body mass index (BMI) between these groups (P = 0.27). Among the women with infertility, the ultrasound examination was altered in 8.5% of the cases (uterine fibroids or adnexal cyst). Among the women with chronic pelvic pain, this was seen in 45.18% of the cases (uterine fibroids or adnexal cyst).

In laparoscopic procedures to treat chronic pelvic pain, 74% of the women presented no alterations, 11% had endometriosis and 15% had adhesions. In the laparoscopic procedures on the infertility group, 47% presented no alterations, 24% had tubal sterilization, 17% had tubal alterations, 5% had endometriosis and 7% had adhesions.

The findings during laparoscopy in the group of women with infertility were: 14.16% with adnexal alterations (simple cyst, endometriomas or adhesions); 19.83%, uterine abnormalities (fibroids, adenomyosis or absence); 18.98% peritoneal alterations (endometriosis or adhesions); and 59.78%, tubal alterations (dilatation and tortuosity, adhesion, tubal sterilization or absence). In the women with chronic pelvic pain, the findings during laparoscopy were: 14.45% with adnexal alterations (simple and hemorrhagic cysts, endometriomas and adhesions); 28.92%, uterine abnormalities (fibroids, adenomyosis or absence); 35.54%, peritoneal alterations (endometriosis or adhesions); and 25.30%, tubal alterations (dilatation and tortuosity, adhesion or tubal sterilization) (Table 1).

**Table 1.**
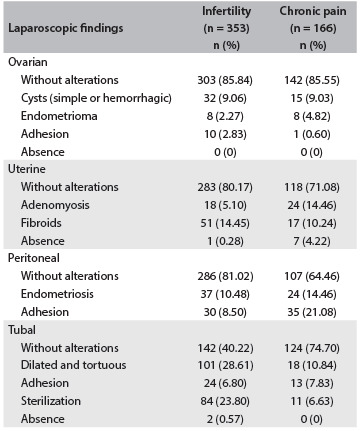
Laparoscopic findings according to the indication

## DISCUSSION

Our study showed that the main indications for diagnostic laparoscopy were infertility (57%) and chronic pelvic pain (27%). Most of the laparoscopic procedures did not show any abnormalities, but the primary findings were tubal alterations in the group of women with infertility and peritoneal alterations (endometriosis or adhesion) in the group of women with chronic pelvic pain.

Similar results can also be seen in the literature. A study on 1654 diagnostic laparoscopic procedures showed that the main indications for the procedure were infertility (98%) and chronic pelvic pain (2%).^2^ Laparoscopy is indicated in 89% of the cases of infertility in the United States, while in Canada it is indicated in 63% of the cases.^3^ In cases of chronic pelvic pain, laparoscopy is indicated in 40%.^4^

Our study showed that in infertility cases, tubal alterations were the most prevalent finding from laparoscopy. In another study on 206 women with infertility, laparoscopy showed that 20.4% had pelvic adhesions, 13.6% tubal obstruction and 5.8% endometriosis.^5^ Another study on 328 infertile women showed that laparoscopy diagnosed that 16% had pelvic adhesions, 19% tubal obstruction, 26% endometriosis and 13% pelvic infection.^6^ In the literature, in cases in which laparoscopy was indicated due to infertility, the main findings were tubal alterations and endometriosis.^3^^,^^7^

Although most of the laparoscopic procedures carried out in the group of women with chronic pelvic pain did not show any abnormalities, the main findings were endometriosis and adhesions. Chronic pelvic pain is characterized by a painful sensation in the lower abdomen or pelvis, which may be either intermittent or constant, with or without a cyclic nature, lasting for at least six months and intense enough to lead the woman to seek medical care. Its prevalence has been estimated as between 12% and 29%.^8^

Laparoscopy in cases of chronic pelvic pain can be useful for diagnosing diseases such as endometriosis, adhesions, ovarian cysts and pelvic inflammatory disease. In cases of endometriosis, laparoscopy is the gold standard for diagnosis, in addition to enable staging (endometriosis grades 1, 2, 3 and 4). Laparoscopy can be used to evaluate subserosal fibroids and differentiate them from ovarian cysts. It can be used to diagnose abnormal uterine findings such as congenital uterine malformations (septate, bicornuate or didelphys uterus), which is not always possible with ultrasound.^1^

In our clinic, 74% of the laparoscopy procedures for investigating for chronic pelvic pain did not present any alterations. This leads us to discuss the importance of better clinical approaches and imaging studies before indicating the procedure. Despite the low complication rate of laparoscopy, it is an invasive method that entails great costs. One of the reasons why laparoscopy might not find any alterations is that abdominal myofascial syndrome might be present, resulting from a change to the abdominal wall, usually secondary to a previous cesarean.

In one study that evaluated 44 women with chronic pelvic pain in comparison with 31 women without pain, laparoscopy found that 88.4% of the group with pelvic pain and 42% of the group without pain presented alterations. In the literature, the incidence of laparoscopic findings among women with chronic pelvic pain was between 35% and 83%.^5^ In a recent study on 85 women with chronic pelvic pain, laparoscopy showed that 20% had pelvic tuberculosis, 13% endometriosis, 9% adhesions and 7% adnexal cysts.^4^

Laparoscopy may be indicated in emergencies, in cases of acute pelvic pain, to identify pelvic inflammatory disease, adnexal torsion, ruptured ectopic pregnancy and ruptured hemorrhagic cysts. It also enables evaluation of the pelvic cavity and the uterus in cases of uterine perforations during surgery or insertion of a intrauterine device.^1^

However, there are still some conditions that limit the use of laparoscopy, due either to a permanent or to a temporary health condition presented by the patient. Such conditions might make it impossible to perform any surgery or might require open surgery because of technical difficulties or because better results are sought. Among these conditions are serious diseases such as heart disease, hemodynamic instability (septic or hypovolemic shock) and severe respiratory diseases, which may worsen through pneumoperitoneum caused by laparoscopy. Intracranial hypertension can also be aggravated by the head-down position in laparoscopy. Other conditions that limit the use of laparoscopy include the presence of distended bowels, which can be damaged by the equipment; presence of a large abdominal mass; advanced pregnancy; histories of several previous surgeries, which might distort the anatomy and hinder viewing; and obesity, which can make it impossible to implement pneumoperitoneum.^1^

Because laparoscopy is a technique that presents little risk, low complication rates and shorter duration of operations, and enables diagnosis and treatment procedures, it has become very important and widely used in the field of gynecology. Thus, knowing what the indications for laparoscopy are, along with the results from this procedure and the profile of the women who would benefit from it, can be beneficial.

Since this study was retrospective, it has limitations due to the lack of data in many records. Thus, we were unable to assess whether differences in socioeconomic level among the women could be a factor interfering in our results. We can infer that the population was homogeneous with regard to economic status, given that our clinic provides healthcare for the general population with low financial power.

## CONCLUSION

The main indications for laparoscopy in gynecology were infertility and chronic pelvic pain. However, in most procedures, no abnormalities justifying these complaints were found. This suggests that there is a need for better clinical research before indicating laparoscopy.
